# SMCNet: State-Space Model for Enhanced Corruption Robustness in 3D Classification

**DOI:** 10.3390/s24237861

**Published:** 2024-12-09

**Authors:** Junhui Li, Bangju Huang, Lei Pan

**Affiliations:** 1 College of Air Traffic Management, Civil Aviation Flight University of China, Deyang 618307, China; junhuili@cafuc.edu.cn; 2 School of Computer Science, Civil Aviation Flight University of China, Deyang 618307, China; leipan.cafuc@hotmail.com

**Keywords:** point cloud, state-space model, object classification, LiDAR, corruption robustness

## Abstract

Accurate classification of three-dimensional (3D) point clouds in real-world environments is often impeded by sensor noise, occlusions, and incomplete data. To overcome these challenges, we propose SMCNet, a robust multimodal framework for 3D point cloud classification. SMCNet combines multi-view projection and neural radiance fields (NeRFs) to generate high-fidelity 2D representations with enhanced texture realism, addressing occlusions and lighting inconsistencies effectively. The Mamba model is further refined within this framework by integrating a depth perception module to capture long-range point interactions and adopting a dual-channel structure to enhance point-wise feature extraction. Fine-tuning adapters for the CLIP and Mamba models are also introduced, significantly improving cross-domain adaptability. Additionally, an intelligent voting mechanism aggregates predictions from multiple viewpoints, ensuring enhanced classification robustness. Comprehensive experiments demonstrate that SMCNet achieves state-of-the-art performance, outperforming the PointNet++ baseline with a 0.5% improvement in mean overall accuracy (mOA) on ModelNet40 and a 7.9% improvement on ScanObjectNN. In corruption resistance, SMCNet reduces the mean corruption error (mCE) by 0.8% on ModelNet40-C and 3.6% on ScanObjectNN-C. These results highlight the effectiveness of SMCNet in tackling real-world classification scenarios with noisy and corrupted data.

## 1. Introduction

In the field of computer vision, three-dimensional (3D) point cloud classification has significantly advanced industries such as autonomous driving, robotics, and augmented reality [1,2,3,4]. Beyond their extensive applications in autonomous driving, 3D technologies have demonstrated remarkable transformative potential across various domains. For example, a spatiotemporal convolutional neural network based on EEG 3D features has been developed to detect driving fatigue, showcasing the utility of 3D data in healthcare and human behavior analysis [5]. Similarly, 3D vision technologies have been employed in a self-developed structural crack damage recognition robot, demonstrating their effectiveness in construction and infrastructure maintenance [6]. In the domain of unmanned aerial vehicles (UAVs), 3D vision has been applied to enhance navigation and operational efficiency in complex environments. For instance, 3D LiDAR-based perception systems enable UAVs to map and navigate through dense forests, urban areas, and industrial settings by accurately detecting obstacles and estimating flight paths. These systems enhance the autonomy and adaptability of UAVs in dynamic and cluttered environments [7].

A core challenge in 3D point cloud processing is the robust recognition and classification of objects within point cloud data. In autonomous driving scenarios, failure to reliably identify vehicles, pedestrians, or other obstacles in point clouds could result in erroneous decision making, potentially leading to accidents. However, real-world data imperfections, sensor inaccuracies, and environmental occlusions exacerbate the difficulty of achieving reliable recognition, further highlighting the need for robustness. Addressing these challenges to ensure accurate and robust point cloud recognition has become a critical focus of recent research.

There are currently two primary strategies for enhancing robustness in 3D point cloud recognition: network architecture design and data augmentation. In recent years, numerous studies have explored innovative architectures to improve the performance of 3D point cloud processing. PointNet [8], a pioneering work, treats point clouds as unordered sets of points, removing the reliance on fixed grid structures and offering a novel approach to point cloud recognition. Subsequent works have adopted strategies such as multi-level feature aggregation, dynamic graph structures, and kernel-based convolutions [9,10,11,12,13,14,15], providing efficient and flexible solutions for point cloud processing. Some studies have also employed attention mechanisms [16,17,18,19] to enhance models’ focus on critical features, allowing them to adaptively learn relationships between different points in the point cloud and improve recognition capabilities. Furthermore, research has explored multi-scale feature extraction methods [20], which extract features at various scales to improve model adaptability to changes in point cloud structure. These network architectures each bring unique innovations to the point cloud domain, offering tailored improvements for different application scenarios and driving continuous progress in point cloud processing.

Data augmentation strategies [21] are a common approach to improving the generalization ability of point cloud recognition models. These include adaptive augmentation for real-world perturbations and benchmarking point cloud classification under various forms of degradation. These strategies modify original data through deformation, mixing, and simulating random loss, scaling, and rotation to mimic the diversity of real-world interference. However, while these methods aim to enhance model generalization, they are not always fully effective since they cannot completely simulate all real-world disturbances. Excessive or inappropriate data augmentation may even degrade model performance.

In recent years, driven by advancements in deep learning, neural radiance fields (NeRFs) have emerged as a groundbreaking method for 3D reconstruction, gaining widespread attention for their exceptional capabilities in high-resolution rendering, photorealistic texture generation, and novel view synthesis [22]. At its core, the method represents a scene as a continuous neural radiance field, encoding color and density values at arbitrary points and directions. By optimizing this implicit representation, NeRFs can effectively learn detailed geometric structures and appearance attributes of a scene from a limited set of multi-view images, subsequently synthesizing high-quality images from novel viewpoints. This approach circumvents the discretization issues commonly encountered in traditional 3D reconstruction methods and exhibits remarkable advantages in handling occlusions and complex lighting conditions [23].

The success of NeRFs has inspired numerous variants, such as Nerfies [24] and NeRF in the Wild [25], which address challenges like high computational costs and dynamic scene reconstruction by introducing deformation fields and refining optimization processes. Compared to traditional multi-view geometry-based 3D reconstruction approaches, the NeRF model and its variants offer several distinct advantages. First, the NeRF model directly learns implicit scene representations from images without requiring explicit geometric modeling, significantly simplifying the reconstruction pipeline. Second, its use of continuous functions enables more natural handling of occlusions and incomplete data, a longstanding limitation of methods based on discrete representations. Finally, the NeRF model ensures high consistency and coherence in novel view synthesis, which is particularly beneficial for applications in virtual reality and augmented reality. Despite these advancements, existing methods still face challenges in handling real-world data imperfections, sensor inaccuracies, and environmental occlusions. For instance, PointNet++ [26] and DGCNN [27] struggle with robustness under various forms of corruption, as demonstrated in benchmarks like ModelNet40-C [28] and ScanObjectNN-C [29]. These benchmarks highlight the need for models that can maintain high performance even in the presence of noise, occlusions, and incomplete data.

To address the challenges of 3D point cloud classification, we propose SMCNet, a novel multimodal framework that introduces innovative solutions to enhance classification accuracy and resilience in real-world scenarios. SMCNet combines multi-view projection and neural radiance fields (NeRFs) to generate high-fidelity 2D representations, effectively mitigating the effects of occlusions and lighting inconsistencies. Compared to traditional methods, the modular architecture and lightweight design of the Mamba model confer higher computational efficiency, making it particularly suitable for resource-constrained environments. Moreover, the Mamba model inherently supports multi-scale feature extraction, offering a natural advantage in capturing the geometric structures and contextual information of complex point cloud data. This study is the first to apply the Mamba model to robustness research in 3D point cloud recognition. Enhancements include a newly designed depth perception module for capturing long-range geometric relationships and a dual-channel structure tailored to strengthen point-wise feature extraction for complex point cloud data. Additionally, SMCNet integrates fine-tuned adapters for the CLIP [30] and Mamba models, enhancing cross-domain adaptability and model generalization. An intelligent voting mechanism aggregates predictions from multiple viewpoints and dynamically adjusts to varying environmental conditions, ensuring robust performance. By leveraging the complementary strengths of the NeRF, CLIP, and Mamba models, SMCNet forms a unified framework that capitalizes on large-scale pre-trained datasets to achieve exceptional recognition capabilities in the presence of noise and imperfections in real-world data. Experimental evaluations validate the outstanding effectiveness of SMCNet across diverse and challenging environments.

The contributions of this paper are summarized as follows:

- We introduce a novel approach within SMCNet that transforms 3D point cloud data into 2D images using multi-view projection and the NeRF model. This method generates high-quality multi-view images that effectively preserve lighting and occlusion effects, enhancing their overall realism.
- To tackle challenges related to local geometric modeling and unidirectional constraints, we present an enhanced Mamba model integrated within SMCNet. By incorporating a depth-perception module to capture long-range dependencies and a dual-channel structure to improve point interactions, we significantly boost the model’s performance.
- To address domain adaptation challenges, we develop an adapter module for fine-tuning both the CLIP and Mamba models within SMCNet. This ensures that the models effectively capture both detailed and abstract representations of point cloud data, thereby improving their processing capabilities.
- We implement an intelligent weighted voting mechanism within SMCNet that adaptively adjusts weights based on model output probabilities and performance metrics from the validation set. This approach enhances the diversity and robustness of the decision-making process. The experimental results demonstrate that our method outperforms baseline approaches.

## 2. Related Work

### 2.1. Network Architectures for Point Clouds

Recent research has focused on designing more expressive and adaptive point cloud encoders and pooling operations to capture essential features. PointNet introduced an innovative approach by treating point clouds as unordered sets of points rather than relying on fixed grid structures, laying the foundation for robust point cloud recognition. Following this, numerous networks specifically designed for point cloud processing have emerged, further advancing the field. Among these, PointNet++ [31] improved performance by introducing a multi-level feature aggregation mechanism. DGCNN (Dynamic Graph CNN) built dynamic graph structures to better capture local features and global contextual information in point clouds. Meanwhile, KPConv [32] (Kernel Point Convolution) employed kernel-based convolution methods, providing an efficient and flexible approach to point cloud processing. PointTransformer introduced self-attention mechanisms, enabling the model to adaptively capture complex interactions between different points in the point cloud, significantly enhancing recognition capabilities. Additionally, the multi-scale feature extraction method, MS-TCN [33], strengthened the model’s sensitivity to changes in point cloud structures by capturing features at various scales. Each of these networks has unique characteristics, offering powerful tools for different scenarios and tasks.

However, Jiachen Sun observed that these networks lack robustness when facing certain complex perturbations. Therefore, he focused on enhancing the robustness of point cloud recognition through the innovation and design of new network architectures. He introduced the first comprehensive benchmark for 3D point cloud corruption robustness, ModelNet40-C, which provides a thorough evaluation of a broad range of representative point cloud models to assess their robustness and generalization. Subsequently, Jie Wang observed that existing point cloud corruption models are primarily derived from CAD models rather than being scanned from real-world objects, which may not adequately reflect the classifier’s performance in realistic scenarios. To assess robustness in real-world conditions, he established a point cloud corruption dataset called ScanObjectNN-C, which more accurately represents real-world corruptions, often characterized by occlusions, biases, and complex backgrounds. However, no network architecture can effectively handle all types of complex disturbances.

### 2.2. Data Augmentation Methods

Data augmentation strategies are crucial in the field of point cloud classification, aiming to improve the model’s generalization ability by simulating real-world perturbations, thereby enhancing the robustness of point cloud recognition.

The PointWOLF method proposed by Sihyeon Kim [34] introduces a localized weighting mechanism to generate diverse data augmentations, aiming to better simulate real-world perturbations. The primary advantage of this approach lies in its ability to effectively preserve the structural integrity of point cloud samples, thereby enhancing model performance in point cloud shape classification tasks. In contrast, Dogyoon Lee’s RSMix method [35] employs a neighborhood function to extract subsets of each sample without disrupting the point cloud structure. This regularization technique significantly improves the performance of deep neural networks on point cloud classification tasks. The neighborhood function in RSMix is utilized to extract local subsets from each point cloud sample, ensuring the retention of structural information. Specifically, the neighborhood can be defined using distance-based K-nearest neighbors (KNN) or spatial partitioning via voxel grids. By carefully selecting neighborhood parameters, such as the number of neighbors (K) or voxel size, the extracted subsets can adequately preserve local structural details. In RSMix, these subsets undergo rigid transformations, such as translation and rotation, before being mixed to generate new training samples. These rigid transformations do not alter the local geometric features of the point clouds, ensuring that the mixed point clouds retain the structural integrity of the original samples. Experimental results and visualizations validate the effectiveness of RSMix in preserving point cloud structure, demonstrating substantial improvements in classification performance and model generalization.

Despite the remarkable improvements in robustness achieved by methods like PointWOLF and RSMix, these approaches are not universally effective. This is because they may not fully replicate the diversity of real-world perturbations, and improper data augmentation can lead to decreased model performance. Therefore, in practical applications, it is essential to carefully consider model performance, task requirements, and data characteristics to select the most suitable data augmentation strategy, ensuring model robustness in complex real-world scenarios.

## 3. Method

### 3.1. Overall

**Figure 1:**
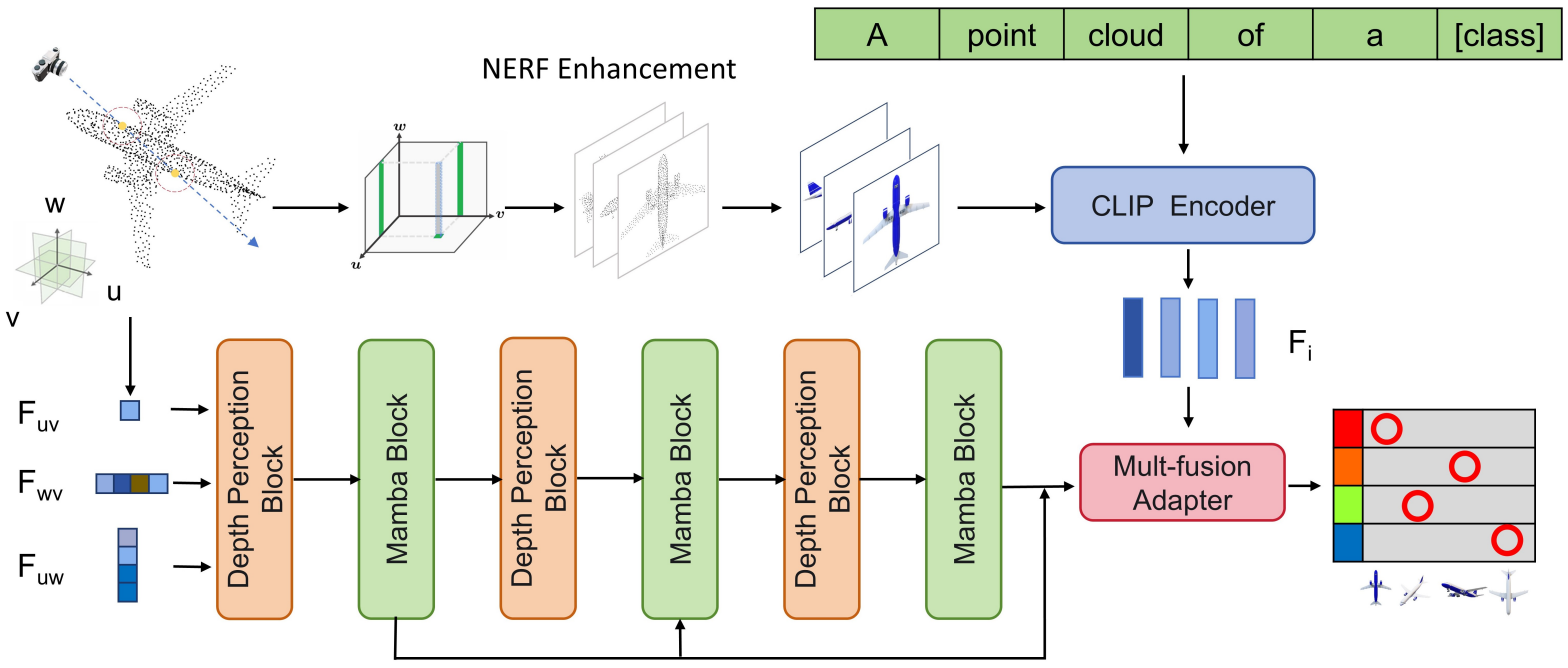
The architecture of the SMCNet: A multimodal 3D point cloud classification framework utilizing CLIP and Mamba.

Traditional 3D point cloud classification methods face challenges such as incomplete data and noise interference. As shown in Figure 1, this study proposes an innovative approach that leverages the CLIP model and the Mamba state-space model to improve the robustness and accuracy of 3D point cloud processing.

Section 3.2 introduces the use of the multi-view projection method and neural radiance fields (NeRFs) to generate high-quality images with realistic textures. Section 3.3 describes the enhanced Mamba model for extracting 3D features. Section 3.4 discusses the use of an adapter to fine-tune CLIP and Mamba. Section 3.5 introduces the integrated intelligent voting mechanism to optimize the classification results, thereby enhancing the system’s decision diversity and robustness.

### 3.2. Neural Radiance Fields for Point Clouds and Multi-View Fusion

In processing 3D point cloud data, because CLIP only accepts text and 2D image inputs, an initial multi-view projection step is required to map 3D point clouds to 2D images. The purpose of this step is to convert the high-dimensional point cloud information into a 2D image representation that can be interpreted by the CLIP model, facilitating subsequent image classification tasks. To generate high-quality multi-view 2D images with realistic lighting principles, we propose a novel approach. First, the 3D object’s point cloud data and multi-view images are captured, which will be used to train the neural radiance field (NeRF) model. Then, a NeRF model is defined, which uses a neural network to predict color values and density from 3D points. The NeRF model is trained using multi-view images and point cloud data to optimize its parameters, enabling it to accurately reconstruct 3D scenes from different viewpoints.

Our proposed method enhances the quality of 3D scene reconstruction and rendering through a novel neural radiance field representation based on point clouds. This method represents the scene as a set of points associated with neural features and confidence scores. Each point is linked to a neural feature vector, and these points are generated using multi-view images and depth multi-view stereo techniques. For each position traversed by a ray, neighboring points within a fixed radius are queried. Each neighboring point contributes its features through an MLP network, and these features are weighted based on distance. Another MLP network computes the volume density for each position, and the final volume density is calculated through weighted aggregation. The formula for aggregated feature computation is as follows:

(1) $$F_{\mathrm{agg}}\left(p\right)=\sum_{i}w_{i}f_{i}$$

where $w_{i}$ is a regulating parameter that controls the attenuation of the distance weights. The view-dependent radiance r(p) is then regressed from the aggregated features and view direction:

(2) $$r\left(p\right)=f_{\mathrm{view}}\left(F_{\mathrm{agg}}\left(p\right),d\right)$$

This is used for subsequent volume rendering. Our rendering process utilizes the aforementioned neural radiance field representation, employing ray marching and volume rendering techniques to generate multi-view images. Along each ray, multiple points in the scene are sampled, and for each sample point, the volume density and radiance are calculated. The radiance contributions of these points are aggregated using the volume rendering equation, producing the final pixel color. The volume rendering equation is as follows:

(3) $$C\left(r\right)=\int_{t_{n}}^{t_{f}}T\left(t\right)\sigma \left(t\right)c\left(t\right)\;dt$$

where T(t) represents the transmittance from $t_{n}$ to $t_{f}$, σ(t) represents the volume density, and c(t) represents the color value.

During training, the lighting model considers the path of light rays through the scene and occlusion relationships, simulating realistic lighting effects. The multi-view 2D images generated by volume rendering ensure that each view adheres to lighting principles. This approach combines neural radiance field technology and lighting models to produce images with high quality, multi-view coherence, and realistic lighting and occlusion effects.

Furthermore, when the quality of the point cloud is low, such as in cases of sparse or incomplete point cloud data, which is common in real-world datasets, this rendering method performs sub-optimally. To address these issues, we propose a simple representation that aggregates point clouds at multiple scale levels and uses sparse voxel grids at different resolutions. To handle point cloud sparsity, we compute averages across multiple scales, but only within those scales where there are enough neighboring points near the ray. The specific formula is as follows:

(4) $$F_{\mathrm{multi}-\mathrm{scale}}\left(p\right)=\sum_{l}\alpha_{l}F_{l}\left(p\right)$$

where *l* represents the scale level, $F_{l}\left(p\right)$ represents the set of neighboring points at position *p* on the *l*-th scale, and $\alpha_{l}$ is the regulating parameter at this scale.

**Figure 2:**
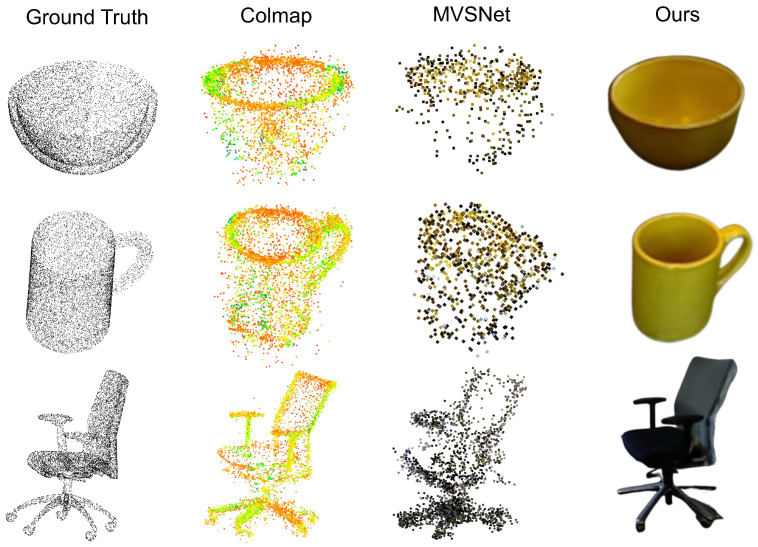
Some examples generated using our method. It is evident that our method generates samples with realistic textures and intricate lighting details.

As illustrated in Figure 2, we compare the results of traditional methods (e.g., Colmap), deep learning-based approaches (e.g., MVSNet), and our proposed method. The comparison highlights the significant advancements achieved by our approach in generating high-quality images for subsequent tasks like image classification and semantic analysis.

Traditional multi-view projection methods, such as Colmap, directly map 3D point cloud data onto a 2D plane without considering lighting effects or occlusion relationships. This often results in sparse, unrealistic representations lacking fine-grained details. Deep learning-based methods like MVSNet partially address these limitations by improving the density and distribution of reconstructed points, yet they still struggle to capture realistic surface textures, lighting effects, and shadows.

Our proposed multi-view information fusion method goes a step further by integrating a sophisticated lighting model. This model accounts for light propagation paths and occlusion relationships, enabling the generation of images with realistic lighting and shadow effects. As shown in the figure, the results generated by our method exhibit superior clarity, smoother surfaces, and a higher degree of realism. This improvement is particularly evident in the finer object contours and the detailed textural representation.

To validate our method’s effectiveness, we applied the same set of inputs to Colmap, MVSNet, and our approach. The qualitative analysis reveals that our method not only outperforms traditional techniques in capturing structural accuracy but also enhances visual realism, making it more suitable for downstream tasks like classification and reconstruction. These results underscore the success of our proposed approach in bridging the gap between 3D data representation and high-quality image generation.

### 3.3. Feature Extraction from 3D Point Clouds

Selecting the appropriate model and method is critical when processing 3D point cloud data. The Mamba model is a novel structured state-space model (SSM) that excels in long-sequence modeling tasks. With its global receptive fields and dynamic weighting mechanisms, Mamba overcomes the limitations of traditional convolutional neural networks (CNNs) and provides advanced modeling capabilities akin to Transformers.

Specifically, Mamba first preprocesses the 3D point cloud data by removing noise and downsampling to reduce data volume. The preprocessing steps include applying filters to eliminate outlier points and anomalies, ensuring data purity and consistency. Downsampling reduces data redundancy by selecting representative points, which helps lower computational complexity and memory requirements.

Once the point cloud is preprocessed, Mamba scans each dimension (X, Y, Z) separately to extract local features in each direction. These features encompass both geometric properties (e.g., point normals, curvature) and statistical characteristics (e.g., density, neighborhood information). By scanning each dimension independently, Mamba is able to capture subtle variations in the point cloud along different axes, thus providing a more comprehensive feature representation.

**Figure 3:**
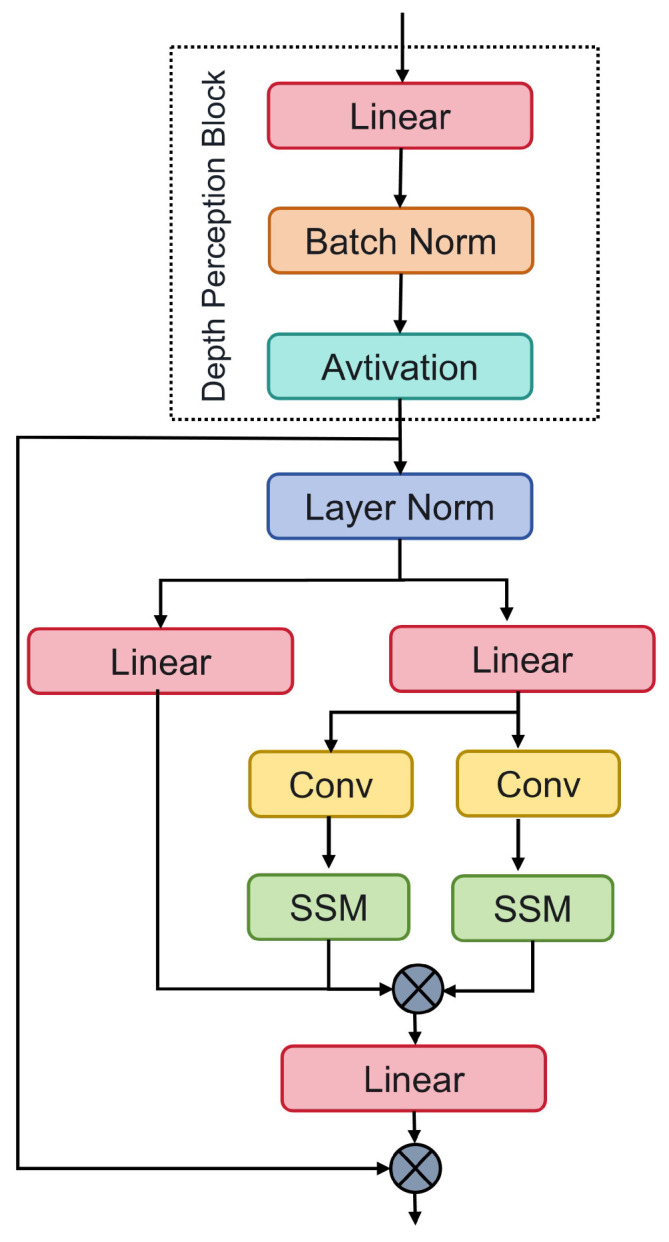
Structural diagram of the improved Mamba model and its depth perception module. This structure improves the accuracy and effectiveness of feature extraction by extracting features from three-dimensional point cloud data, combining global receptive fields and dynamic weighting mechanisms.

However, existing Mamba models face certain limitations in local geometric modeling and unidirectional processing. Local geometric modeling may fail to capture the global structural features of the point cloud, and unidirectional processing may overlook the interactions and relationships between different dimensions. To address these issues, we introduce a depth-perception module, as illustrated in Figure 3, consisting of linear layers, batch normalization layers, and activation functions. This module is specifically designed to enhance the extraction of relevant features from the point cloud data.

The proposed architecture connects three depth-perception modules in series with the Mamba module. This improved Mamba model enhances both local geometric modeling and unidirectional processing. The combination of linear layers, batch normalization, and activation functions increases the diversity and complexity of the feature representations, while batch normalization improves the stability, and activation functions introduce the necessary nonlinearity to the feature extraction process.

While processing these features, Mamba captures long-range dependencies through its global receptive field, allowing the model to understand the point cloud data over a larger spatial scope. To further enhance this capability, we introduce a dual-channel structure that enables each point to interact with any other point in the scene. This facilitates the identification of associations between distant points, improving the coherence and consistency of the extracted features. Additionally, the dynamic weighting mechanism allows the model to adjust the importance of different features during processing, based on their relevance to the task at hand. This ensures that critical features are emphasized, leading to more precise and effective feature extraction.

Through this scanning and processing pipeline, each point cloud generates independent feature vectors for each of the X, Y, and Z dimensions. These feature vectors are ultimately combined into a comprehensive 3D feature matrix, which preserves the spatial structure of the point cloud while effectively fusing features from all three dimensions. This high-dimensional feature space serves as a rich input for subsequent classification and recognition tasks, enabling more accurate and robust point cloud analysis.

### 3.4. Adapter Fine-Tuning

CLIP, designed based on the Vision Transformer, demonstrates exceptional performance in processing image data. However, when dealing with the geometric information inherent in point cloud data, CLIP may not be as effective as models specifically designed for point cloud processing. Projecting the geometric structure of point clouds into images may result in the loss of some information, which negatively impacts classification tasks. To address this, we introduce an adapter mechanism, incorporating additional small networks to fine-tune both CLIP and Mamba simultaneously, thereby preserving and better handling the geometric information in point clouds.

Adapters are specially designed network components that can be inserted into existing models to adapt them to new input data types or tasks. This method allows the model to transfer knowledge from image tasks to point cloud tasks while retaining its learned capabilities in image processing, thus addressing domain adaptation challenges. The adapter design enables the model to better adjust to the data of the new domain. After pre-training the model on 2D tasks, the adapter helps the model learn the specific characteristics of the new 3D domain, improving its performance on the new task.

In this study, the Mamba encoder is used to process the 3D features of the point cloud, which are flattened and then fused with the 2D features from CLIP’s image encoder and the textual information from the CLIP text encoder. To avoid potential inconsistencies and high-dimensionality issues that may arise from direct concatenation, feature fusion methods such as weighted averaging or attention mechanisms are employed. These methods ensure consistency and effectiveness in the common feature space between 3D, 2D, and textual information. Subsequently, the fused feature vectors undergo adapter-based fine-tuning.

**Figure 4:**
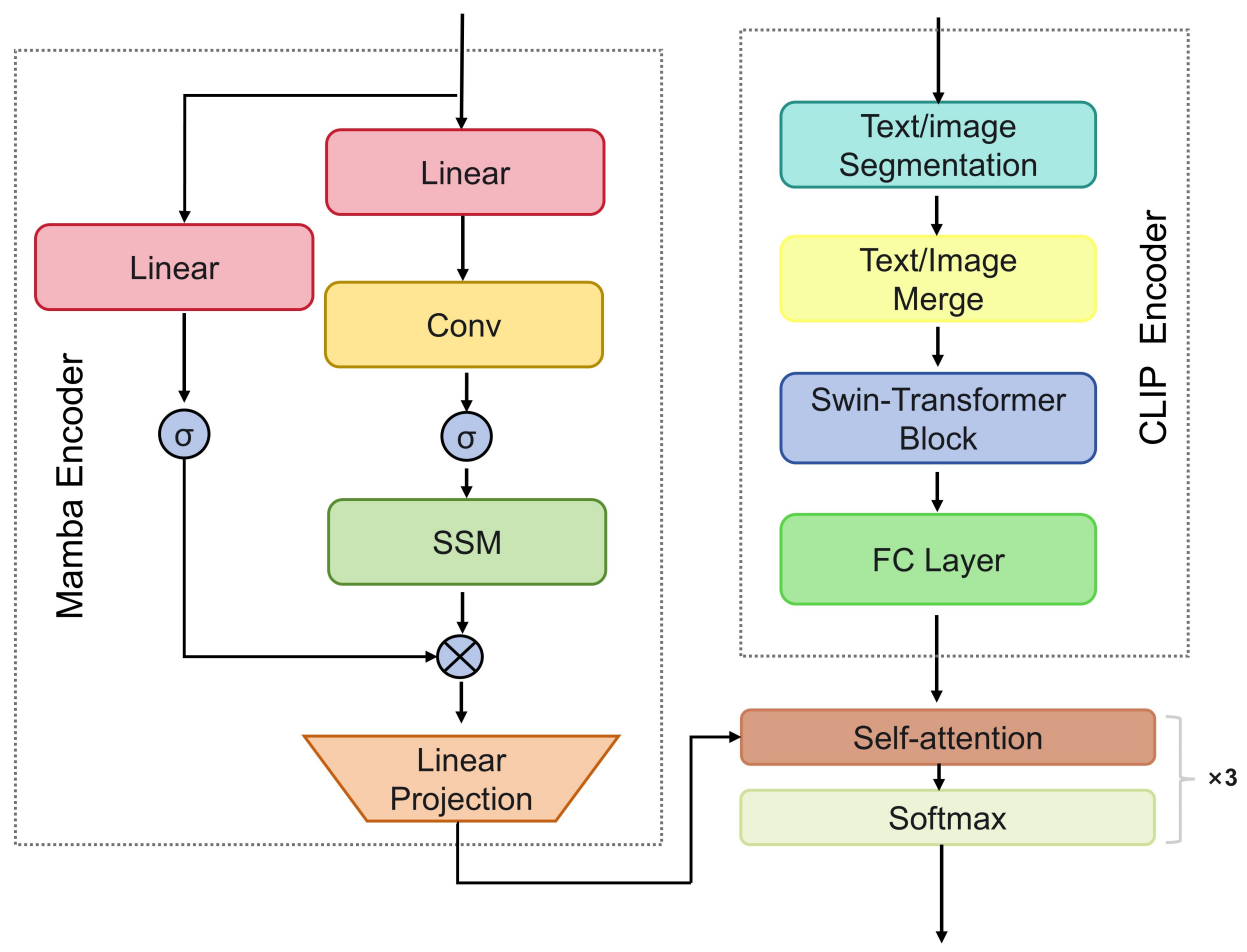
The architecture of the improved adapter. It effectively integrates information from different dimensions through the introduction of linear projection and convolutional layers. In addition, the adapter injects processed 3D features after the output of each Swin Transformer layer, thereby enhancing the model’s ability to capture details and abstract representations of point cloud data.

As shown in Figure 4, we introduce additional small networks to fine-tune both the CLIP and Mamba encoders. After the Mamba encoder’s output, linear projection and convolutional layers are introduced to process and fuse 3D features. These processed 3D features are then integrated with the 2D features from CLIP’s image encoder. This process allows the adapter to inject processed 3D features after the output of each Swin Transformer layer, ensuring the model captures the details and abstract representations of point cloud data more comprehensively.

During the adapter fine-tuning process, we also integrate contrastive learning techniques. Contrastive learning enhances the model’s discriminative power for 3D point cloud features by constructing positive and negative sample pairs. Specifically, a training set is generated during training that contains both similar and dissimilar sample pairs. Similar pairs consist of point cloud data from different views or similar poses of the same object, while dissimilar pairs are formed from different objects or point cloud data from widely different perspectives. By maximizing the similarity of positive pairs in feature space and minimizing the similarity of negative pairs, the model effectively learns discriminative features from the point cloud data.

Since point cloud data differ significantly from image data in their representation, using adapters helps reduce the dimensional mapping gap. Furthermore, adapters can learn more complex nonlinear relationships within the point cloud data, allowing better adaptation to the complex structures and features inherent in the input data. By applying domain adaptation strategies, we aim to optimize the 3D-to-2D mapping, significantly enhancing the model’s robustness and generalization performance in point cloud classification tasks.

Through this adapter fine-tuning approach, we not only retain CLIP’s advantage in processing image data but also enhance its ability to handle 3D point cloud data. This innovative adapter design and fine-tuning strategy enable the model to extract and classify features more effectively when faced with different data types, significantly improving its performance across various application scenarios.

### 3.5. Integration of 2D and 3D Knowledge

To achieve higher accuracy and robustness in 3D point cloud classification tasks, this study introduces an improved mechanism based on intelligent voting. The purpose of this mechanism is to effectively integrate the outputs from multiple CLIP models by assigning weighted “votes” to each model’s output for every category. The weighting process is dependent on each model’s output probability or score for a specific category, combined with its overall performance on the validation set. This enables dynamic confidence weight adjustments, ensuring that models with superior performance have a greater influence on the final classification decision. The mechanism considers both the individual performance of each model and the consistency of predictions across models, as well as potential correlations between different categories.

In the intelligent voting process, the total vote count for each category is calculated by summing the weighted votes from all CLIP models for that category, as defined by the following equation:

(5) $$V_{i}=\sum_{j=1}^{N}w_{j}s_{ij}$$

where $w_{j}$ is the weight of the *j*-th CLIP model, determined by its overall performance on the validation set. $s_{ij}$ represents the output probability or score of the *j*-th CLIP model for the *i*-th category, and *N* denotes the total number of CLIP models. The final classification decision is made based on the category $C_{\max}$ that receives the highest total vote count, expressed as

(6) $$C_{\max}=\arg\max_{i}V_{i}$$

Compared to traditional majority voting or averaging mechanisms, the improved voting mechanism places greater emphasis on the quality and consistency of model outputs. By intelligently assigning weights, the mechanism can more accurately reflect each model’s reliability for specific classification tasks, resulting in superior performance when combining the outputs of multiple models. This intelligent integration of multiple models’ outputs effectively reduces the impact of individual model biases on the final classification results, thereby enhancing the accuracy of identifying complex 3D scenes.

Additionally, by considering the significant differences in predictions made by different models for the same category, the intelligent weight allocation mechanism mitigates the influence of outlier models, improving overall classification consistency and system robustness. This innovative approach ensures that the combined output leverages the strengths of all contributing models, leading to a more robust and accurate classification system.

### 3.6. Loss Function

To better optimize the model and avoid local minima, this study adopts a cosine learning rate scheduler during fine-tuning. The cosine scheduler adjusts the learning rate according to the shape of the cosine function, ensuring a higher learning rate in the early stages of training and a gradual decrease towards the later stages. This helps the model optimize more robustly and improves its performance on fine-tuning tasks. The cosine learning rate update rule is as follows:

(7) $$\eta_{t}=\eta_{\min}+\frac{1}{2}\left(\eta_{\max}-\eta_{\min}\right)\left(1+\cos\left(\frac{t}{T}\pi \right)\right)$$

where $\eta_{t}$ is the current learning rate, $\eta_{\min}$ is the minimum learning rate, $\eta_{\max}$ is the initial learning rate, *t* is the current training epoch, and *T* is the total number of training epochs.

Additionally, this paper employs a smoothed loss calculation method, using exponential moving averages to smooth out loss fluctuations. Smoothed loss helps provide a clearer understanding of model performance trends during training, preventing large oscillations in loss values caused by the randomness of individual iterations. The smoothed loss is calculated as follows:

(8) $$L_{\mathrm{smooth}}^{\left(t\right)}=\beta L_{\mathrm{smooth}}^{\left(t-1\right)}+\left(1-\beta \right)L_{t}$$

where $L_{\mathrm{smooth}}^{\left(t\right)}$ is the updated smoothed loss, $L_{\mathrm{smooth}}^{\left(t-1\right)}$ is the smoothed loss from the previous iteration, $L_{t}$ is the current iteration loss, and β is the smoothing factor, typically set to a small value, such as 0.9.

By utilizing a cosine learning rate scheduler and smoothed loss calculations, the model is better optimized during fine-tuning, resulting in improved convergence speed and overall performance.

## 4. Experiments

To validate the effectiveness of our approach, we conducted comparative experiments on two common point cloud datasets: ScanObjectNN [36] and ModelNet40 [37]. Furthermore, we tested the corruption robustness of the model on the corrupted point cloud datasets ModelNet40-C and ScanObjectNN-C.

**Datasets.** The datasets used in the experiments include the ScanObjectNN, ModelNet40, ModelNet40-C, and ScanObjectNN-C datasets. ScanObjectNN, released in 2019 by The Hong Kong University of Science and Technology, is a public dataset containing 15 object categories. Each sample consists of point cloud data obtained by scanning occluded objects from the real world, with some samples exhibiting background occlusion or deformation, making this dataset more challenging compared to others. The dataset includes a total of 2902 point cloud samples, with 2321 samples in the training set and 581 samples in the test set.

ModelNet40 is a classic point cloud public dataset introduced by Princeton University, containing 40 object categories. Each sample is a clean, manually sampled dataset, with a total of 12,311 samples, of which 9843 are used for training and 2468 for testing.

ModelNet40-C, introduced by Jiachen Sun et al. from the University of Michigan in 2022, is a public dataset simulating corruption types in real-world scenarios on the ModelNet40 test set. Each sample has undergone morphological transformations, noise addition, and density changes, representing three types of corruptions. These transformations include five different categories, resulting in a total of 15 corruption types.

**Figure 5:**
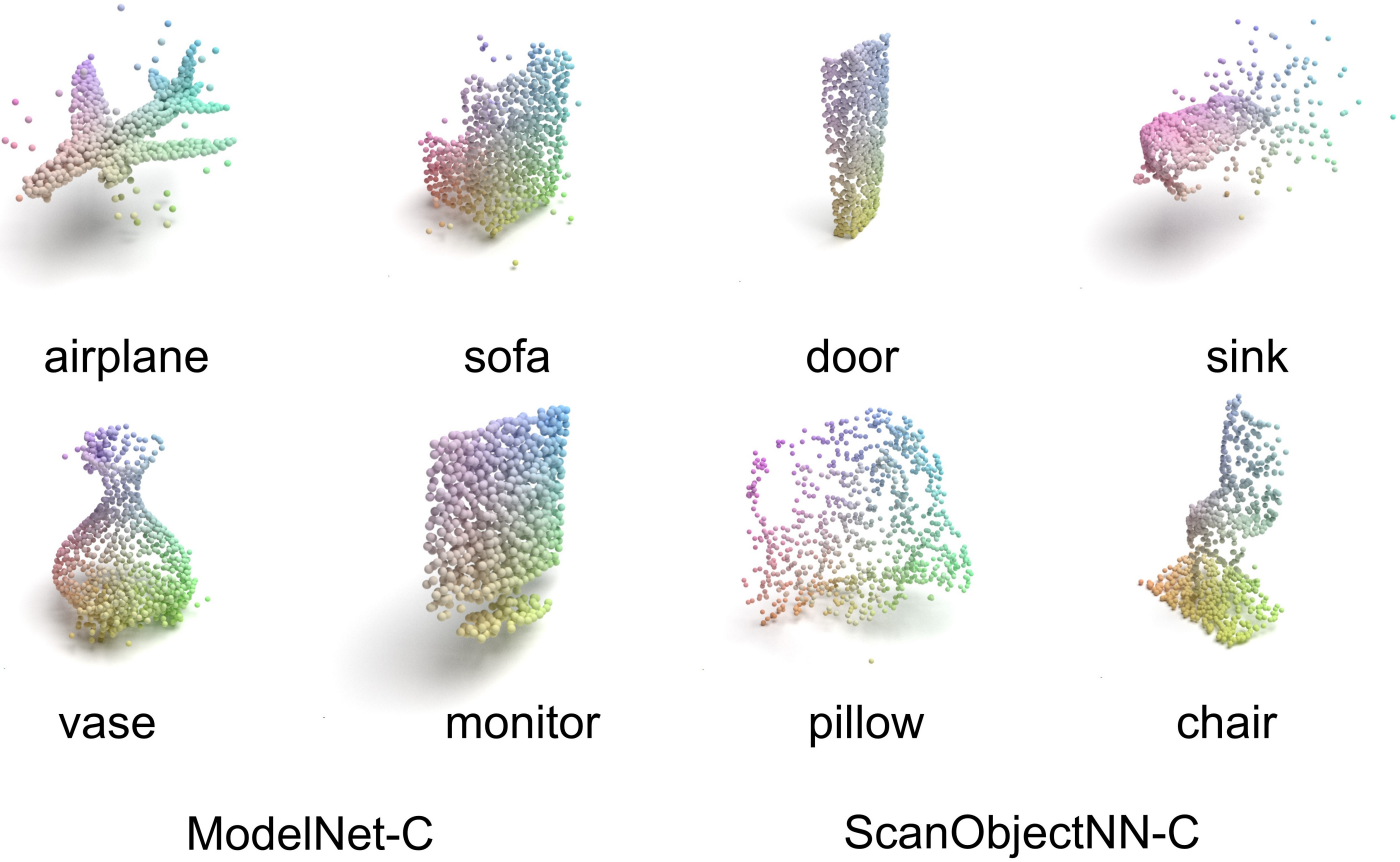
Some samples in ModelNet-C and ScanObjectNN-C.

The ScanObjectNN-C dataset is derived from real-world scans, making it more representative of actual corruption scenarios. Specifically, objects in ModelNet-C originate from CAD models, whereas samples in ScanObjectNN-C are captured from real environments, often exhibiting occlusions, partial views, and complex backgrounds. Figure 5 shows some samples in ModelNet-C and ScanObjectNN-C.

**Evaluation metrics.** We adopt overall accuracy (OA) as the primary metric for evaluating the performance of point cloud classification tasks. OA is a comprehensive indicator that measures the accuracy of the classification model across all categories, calculated as the proportion of correctly classified point clouds to the total number of point clouds. A higher OA value indicates better overall classification performance.

For evaluating ModelNet40-C, we use the mean corruption error (mCE). To normalize the severity of different corruptions, mCE is computed using the classic point cloud classification method DGCNN as the baseline. The formula for mCE is as follows:

(9) $$CE_{l}=\frac{1-OA_{l}}{1-OA_{\mathrm{baseline}}}$$

where $OA_{l}$ is the overall accuracy on the corruption test set of corruption level *l*, and $OA_{\mathrm{baseline}}$ is the overall accuracy of the baseline model. The mCE is the mean of CE over all seven corruption types:

(10) $$mCE=\frac{1}{N}\sum_{l=1}^{N}CE_{l}$$

where N=7 is the total number of corruption types.

**Experimental Setup.** The experiments were conducted in the following environment: Python 3.8, PyTorch 1.11.0, two GTX 3090 GPUs, CUDA 11.3, and Ubuntu 20.04. The experimental parameters were as follows: the number of epochs was set to 100, batch size to 2, and the initial learning rate to 0.01. Stochastic gradient descent (SGD) with momentum of 0.9 was used as the optimizer. We also applied a cosine learning rate scheduler to decay the learning rate and smoothed the loss function.

### 4.1. Experimental Results

**ModelNet40 and ScanObjectNN.** As shown in Table 1, on the ModelNet40 dataset our method achieved an overall accuracy (OA) of 92.7% and a mean accuracy (mAcc) of 92.5%. These results indicate that the proposed method can effectively handle synthetic and clean point cloud data. Although the OA is slightly lower than the state-of-the-art PointMLP (which achieves 94.5% OA and 91.4% mAcc), our method still demonstrates strong performance. Specifically, our method improves the average classification accuracy by 7.86 percentage points compared to the baseline network PointNet++ (which has an mAcc of 86.0%).

On the more challenging ScanObjectNN dataset, our method achieved an OA of 85.8% and a mAcc of 83.2%. These results slightly outperform the Point-TNT method (which has an OA of 79.2% and mAcc of 75.8%) and are competitive with the state-of-the-art PointMLP (which achieves 85.4% OA and 83.9% mAcc). This demonstrates that our method exhibits strong classification performance in the face of real-world variations and noisy data.

In terms of model complexity, our method has 55.1 million parameters and requires 10.5 GFLOPs. While this is higher than some lightweight models like PointNet++ (1.41M params, 2.5 GFLOPs), it is competitive with other high-performance models such as Point-BERT (63.1M params, 12.5 GFLOPs). The increased complexity is justified by the improved performance on both synthetic and real-world datasets.

**ModelNet-C and ScanObjectNN-C.** Comprehensive evaluations on the ModelNet-C and ScanObjectNN-C datasets, along with an in-depth analysis of different noise levels and densities (Table 2, Table 3 and Table 4), demonstrate the robustness and generalizability of our proposed model.

On the ModelNet-C dataset, as shown in Table 2, our method achieves an mCE score of 85.5%, outperforming existing baseline methods such as PointNet++ (107.2%) and RPC (86.3%). Compared to state-of-the-art methods, our model consistently demonstrates strong performance across multiple corruption types, particularly in handling scaling (Sca, 100.1%), jitter (Jit, 70.7%), global point dropout (Drop-G, 79.1%), and local point dropout (Drop-L, 69.8%). Furthermore, integrating the PointWOLF and RSMix strategies further enhances our model’s robustness, reducing the mCE score to 79.4% and 86.7%, respectively.

On the ScanObjectNN-C dataset (Table 3), our model achieves an mCE score of 88.5%, highlighting its resilience against real-world perturbations. The performance remains consistent across different types of distortions, with notable improvements under the PointWOLF (mCE = 86.7%) and RSMix (mCE = 86.7%) strategies. These results confirm that our method not only achieves robustness but also maintains high classification accuracy under diverse and challenging corruption scenarios.

To further analyze the generalizability of our model, Table 4 evaluates its performance on the full ModelNet-C dataset across varying noise levels and densities. As the severity of noise decreases or the density of the point cloud increases, the accuracy consistently improves, with the model achieving an average accuracy of 100.10% on scaling (Sca) and robust performance across jitter (70.7%), global dropout (79.1%), and local dropout (69.8%). This comprehensive evaluation highlights the adaptability of our model to diverse point cloud features, providing valuable insights into its practical applicability in real-world scenarios with varying noise levels and densities.

### 4.2. Ablation Studies

**Figure 6:**
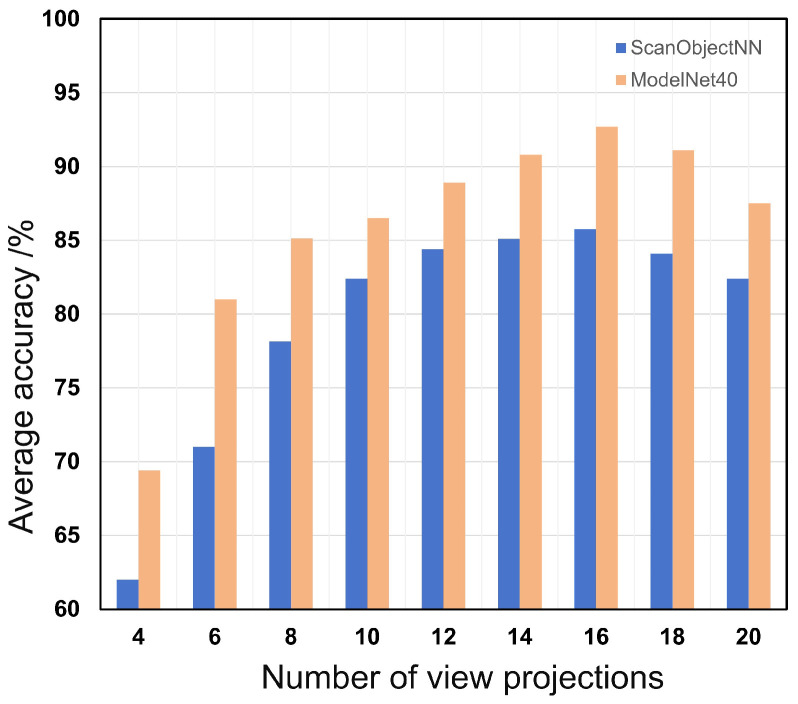
Ablation study on the impact of number of projections on 3D point cloud classification model performance. Blue: ScanObjectiNN; orange: ModelNet40.

**Validation of view projection numbers.** In this ablation study, we systematically investigate the impact of the number of projections on the performance of the 3D point cloud classification model. Through multiple experiments on the ModelNet40 and ScanObjectiNN datasets, we experimented with the number of projections set to 4, 6, 8, 10, 12, 14, 16, 18, and 20, as shown in Figure 6.

The results on the ScanObjectiNN dataset show that through 4 projections, the classification accuracy of the model is 72.4%. As the number of projections increases, the model’s performance also improves. The model performs best with 16 projections, achieving an accuracy of 85.8%. However, beyond 16 projections, the model shows signs of overfitting. This indicates that, for our 3D point cloud classification task, using 16 projection angles yields optimal performance.

**Performance verification of the adapter module.** This experiment aims to evaluate the performance of the adapter module under different network structures and determine the optimal adapter configuration. The experiments were conducted on the ModelNet-C dataset, and the results are shown in Table 5. We compared two mainstream network structures: fully connected layers and self-attention layers. For each structure, three experiments were conducted to assess the impact of different layer configurations on the model’s performance.

The results indicate that for the fully connected layer structure the two-layer fully connected adapter structure exhibited the best performance, with an mCE of 85.5% and an OA of 84.8%. As the number of layers increased, the difficulty of optimization also increased. Although deeper networks contain more parameters, they may lead to overfitting. The two-layer fully connected structure provided sufficient parameters to learn the complex characteristics of the data while avoiding excessive complexity, which helped the model generalize better to unseen data.

In contrast, the self-attention layer structure underperformed across all configurations compared to the fully connected structure. The fully connected structure demonstrated higher parameter and computational efficiency in the adapter module, enabling the model to quickly adapt to new tasks and reduce the risk of overfitting. Additionally, the simplicity of the fully connected structure facilitated improved model training and generalization.

**Validation of the Mamba module’s performance.** The objective of this experiment is to assess the performance of the Mamba model under various architectural configurations. The evaluation was conducted on the ModelNet-C dataset, and the results are presented in Table 6. The findings indicate that the original Mamba module exhibits a misclassification error index (mCE) of 88.4% with an overall classification accuracy (OA) of 78.1%. Upon the integration of the depth perception module, the mCE slightly increased to 85.8%, yet the OA notably improved to 84.4%, signifying the depth perception module’s contribution to enhancing classification accuracy. Subsequently, when the dual-channel structure was introduced, the mCE was recorded at 86.7% with an OA of 82.6%, indicating that the dual-channel structure also marginally improves classification performance, albeit less effectively than the depth perception module alone. When both the depth perception module and the dual-channel structure were concurrently incorporated, the mCE of the Mamba module was 85.5%, comparable to that of the module with the depth perception module alone, but the OA was elevated to 84.8%, surpassing the use of either modification in isolation. This suggests that the combination of the two enhancements not only maintains a lower mCE but also exhibits a synergistic effect in boosting OA, thereby conferring a more comprehensive performance improvement to the Mamba module. In summary, the joint utilization of the depth perception module and the dual-channel structure yields superior classification performance for the Mamba module.

**Performance verification of different branches.** Through ablation experiments, we systematically evaluated the performance of different methods on the 3D point cloud classification task, as shown in Table 7. First, using the direct projection method, the system achieved a mean corruption error (mCE) of 1.012 and an overall accuracy (OA) of 77.4%. This represents the most basic implementation, showing preliminary classification performance.

When the neural radiance fields (NeRFs) and multi-view fusion were introduced, the results improved significantly. Specifically, the mCE was greatly reduced to 86.7%, while the OA increased to 81.2%. This improvement indicates that NeRF projections provide significant advantages in generating high-quality, multi-view 2D images, helping to capture more comprehensive scene information and thus improving classification accuracy.

Further, combining NeRF projections with textual information led to an additional reduction in mCE to 86.1%, and OA improved to 83.5%. This stage of improvement highlights the importance of textual information in enriching image descriptions and enhancing classification features. By integrating multimodal information, the classification system can more accurately identify and classify different objects.

Finally, after introducing the state-space model Mamba, the system’s performance improved once again, with the mCE dropping to 85.5% and the OA reaching 84.8%. The incorporation of Mamba enabled the system to capture more complex long-range dependencies, further enhancing feature integrity and consistency. This demonstrates the significance of Mamba in handling 3D point cloud data, as its global receptive field and dynamic weighting mechanism allow the system to better address real-world complex point cloud data.

In summary, the ablation results clearly demonstrate the significant contributions of each module to the system’s robustness and classification performance. The gradual introduction of NeRF projections, textual information, and the Mamba module sequentially enhanced the system’s performance, validating the effectiveness and innovation of our multimodal 3D point cloud classification algorithm.

**Limitation analysis.** The SMCNet framework demonstrates excellent robustness in addressing the issue of 3D point cloud data classification under corruption, but it also has certain limitations and areas for improvement. Firstly, the generation of high-quality 2D representations in SMCNet heavily relies on multi-view projection and neural radiance fields (NeRFs). If the input point cloud data are of poor quality, unevenly distributed, or if the view selection is inappropriate, it may lead to the loss of key features, ultimately affecting classification accuracy. Additionally, the computational complexity of NeRFs is high, especially when processing high-resolution scenes. This poses a significant challenge for deployment on resource-constrained hardware, limiting the framework’s feasibility in real-time applications. Secondly, the current evaluation of SMCNet primarily depends on standard datasets such as ModelNet40-C and ScanObjectNN-C. While these datasets cover a certain range of corruption types, they do not fully reflect the diversity and randomness of real-world complex environments. Factors such as extreme weather conditions, dynamic object occlusions, and sensor noise can have a greater impact on model performance, but these have not been adequately simulated in the experiments.

Future research should focus on optimizing the overall computational efficiency of SMCNet. This includes designing lightweight network architectures, introducing more efficient feature extraction methods, and exploring quantization and pruning techniques to reduce model complexity. To address the complexities of real-world applications, the robustness of the model can be further extended through dynamic adaptive mechanisms or online learning methods, enabling the model to adjust its adaptation to new environments during runtime. Additionally, designing more comprehensive corruption scenario datasets and integrating multimodal information (such as RGB-D data or IMU data) will help enhance the model’s robustness and generalization capabilities in diverse scenarios.

The significance of this research extends beyond improving the accuracy and stability of 3D point cloud classification. It brings substantial value to multiple fields. For instance, in autonomous driving, enhanced robustness can improve environmental perception safety. In intelligent robotics, improved robustness and real-time performance can support efficient operations in complex task scenarios. In smart cities and infrastructure maintenance, the expanded application of 3D vision technology can significantly enhance work efficiency and intelligence levels. Therefore, further optimization and expansion of the SMCNet framework are not only important for academic research but also provide solid technical support for the development of practical applications across multiple domains.

## 5. Conclusions

In this study, we have presented a novel 3D point cloud recognition algorithm, SMCNet, that synergistically combines the strengths of the CLIP and Mamba models to tackle the challenges inherent in real-world 3D point cloud classification. By utilizing multi-view projection and neural radiance fields (NeRFs), our approach generates high-quality, multi-angle 2D images that effectively capture the comprehensive scene information. The integration of the CLIP model for image classification alongside the enhanced Mamba model for 3D feature extraction, complemented by an adapter module for fine-tuning, significantly improves domain adaptation performance.

Moreover, our proposed intelligent voting mechanism refines the classification process by performing weighted fusion of output labels from multiple views, thereby enhancing system robustness. Experimental results on the ModelNet40, ScanObjectNN, and ModelNet40-C datasets clearly demonstrate the superiority of SMCNet over existing benchmark networks. This work not only provides valuable insights into the advancement of 3D point cloud classification but also establishes a solid foundation for further enhancing the robustness and accuracy of 3D point cloud recognition systems.

## Figures and Tables

**Table 1:** Comparison of 3D point cloud classification accuracy on ModelNet40 and ScanObjectNN datasets.

Method	ModelNet40		ScanObjectNN		Params	GFLOPs
	OA	mAcc	OA	mAcc		
PointNet	89.2	86.0	68.2	63.4	3.5M	1.2
PointNet++	92.2	91.4	77.9	75.4	1.4M	2.5
DGCNN	92.9	90.2	78.1	73.6	1.7M	3.8
PointCNN	92.2	88.1	78.5	75.1	1.5M	2.3
GBNet [38]	93.8	91.0	80.5	77.8	8.4M	4.2
PRA-Net [39]	93.7	91.2	82.1	79.1	1.8M	3.1
Point-TNT [40]	92.6	-	83.5	81.0	1.5M	2.8
Point-BERT	93.8	93.8	83.5	83.1	63.1M	12.5
PointMLP [41]	94.5	91.4	85.4	83.9	12.6M	5.0
Ours	92.7	92.5	85.8	83.2	55.1M	10.5

**Table 2:** Robustness evaluation of 3D point cloud classification method on ModelNet-C corrupt dataset.

Method	mCE (↓)	Sca	Jit	Drop-G	Drop-L	Add-G	Add-L	Rot
DGCNN	100.0	100.0	100.0	100.0	100.0	100.0	100.0	100.0
PointNet	142.2	126.6	64.2	50.0	107.2	298.0	159.3	190.2
RSCNN [42]	113.0	107.4	117.1	80.6	151.7	71.2	115.3	147.9
SimpleView [43]	104.7	87.2	71.5	124.2	135.7	98.3	84.4	131.6
GDANet [44]	89.2	83.0	83.9	79.4	89.4	87.1	103.6	98.1
PointMLP	90.5	85.0	87.0	90.0	92.0	88.0	91.0	93.0
Point-BERT	124.8	93.6	125.9	69.0	115.0	193.2	144.0	132.6
CurveNet [45]	92.7	87.2	72.5	71.0	102.4	134.6	100.0	80.9
PAConv	110.4	90.4	146.5	100.0	100.5	108.5	129.8	96.7
PointNet++	107.2	87.2	117.7	64.1	180.2	61.4	99.3	140.5
RPC	86.3	84.0	89.2	49.2	79.7	92.9	101.1	107.9
Ours	85.5	100.1	70.7	79.1	69.8	79.7	93.3	79.7
PointNet++ (PointWOLF)	82.5	81.9	135.1	67.3	130.4	43.1	68.4	51.2
PointNet++ (RSMix)	86.3	89.4	164.9	48.4	73.9	26.1	32.7	168.4
Ours (PointWOLF)	79.4	82.2	96.2	68.0	76.1	67.0	76.6	89.6

**Table 3:** Robustness evaluation of 3D point cloud classification method on ScanObjectNN-C corrupt dataset.

Method	mCE (↓)	Sca	Jit	Drop-G	Drop-L	Add-G	Add-L	Rot
DGCNN	100.0	100.0	100.0	100.0	100.0	100.0	100.0	100.0
PointNet++	96.9	89.7	110.3	55.0	127.7	94.7	90.5	110.7
RPC	132.6	131.7	107.3	145.5	130.5	114.2	158.7	140.2
PointNeXt	92.1	80.3	107.9	80.7	94.2	94.4	87.5	99.5
Ours	88.5	83.7	101.7	79.1	83.8	96.7	82.3	99.7
PointNet++ (PointWOLF)	96.4	84.0	108.7	70.5	156.6	87.7	90.9	76.1
PointNet++ (RSMix)	91.9	89.0	100.7	55.6	99.0	94.6	89.1	114.9
Ours (RSMix)	86.7	81.0	98.5	76.7	81.1	93.5	79.7	96.4

**Table 4:** Robustness evaluation of our method on the full ModelNet-C dataset with different point cloud features (noise levels and densities).

Level	Sca	Jit	Drop-G	Drop-L	Add-G	Add-L
1	77.1	57.5	63.0	57.2	63.8	73.0
2	87.1	63.6	69.6	61.5	70.2	80.6
3	99.7	69.5	78.6	68.1	79.1	91.8
4	111.4	76.4	86.7	75.5	86.8	104.1
5	125.3	86.4	97.4	86.6	98.6	116.9
Avg	100.1	70.7	79.1	69.8	79.7	93.3

**Table 5:** Ablation study on performance comparison of ModelNet-C dataset using adapter module configuration.

Method	Number	mCE (↓ )	OA
Fully connected	1	87.5	85.1
	2	85.5	84.8
	3	91.4	84.1
Self-attention	1	90.3	83.6
	2	86.7	85.0
	3	92.2	83.0

**Table 6:** Ablation study of Mamba module variants on performance metrics using the ModelNet-C dataset.

Method	mCE (↓)	OA
Original Mamba	88.4	78.1
Mamba + Depth Perception Module	85.8	84.4
Mamba + Dual Channel Structure	86.7	82.6
Mamba + Depth Perception Module + Dual Channel Structure	85.5	84.8

**Table 7:** Comparative analysis of ablation study results for enhancing 3D point cloud classification accuracy with various methodological components.

DP	NeRF	Text	Mamba	mCE (↓)	OA
✓				100.0	77.4
	✓			86.7	81.2
	✓	✓		86.1	83.5
	✓	✓	✓	85.5	84.8

## Data Availability

The data presented in this research are available upon request from the corresponding author.
